# Establishment and Characterization of a Buffalo (*Bubalus bubalis*) Mammary Epithelial Cell Line

**DOI:** 10.1371/journal.pone.0040469

**Published:** 2012-07-09

**Authors:** Vijay Anand, Nilambra Dogra, Surender Singh, Sudarshan N. Kumar, Manoj K. Jena, Dhruba Malakar, Ajay K. Dang, Bishnu P. Mishra, Tapas K. Mukhopadhyay, Jai K. Kaushik, Ashok K. Mohanty

**Affiliations:** 1 National Dairy Research Institute (NDRI), Karnal, India; 2 National Centre for Human Genome Studies and Research (NCHGSR), Punjab University, Chandigarh, India; 3 Indian Veterinary Research Institute (IVRI), Izatnagar, India; INRA, UR1282, France

## Abstract

**Background:**

The objective of this study was to establish the buffalo mammary epithelial cell line (BuMEC) and characterize its mammary specific functions.

**Methodology:**

Buffalo mammary tissue collected from the slaughter house was processed enzymatically to obtain a heterogenous population of cells containing both epithelial and fibroblasts cells. Epithelial cells were purified by selective trypsinization and were grown in a plastic substratum. The purified mammary epithelial cells (MECs) after several passages were characterized for mammary specific functions by immunocytochemistry, RT-PCR and western blot.

**Principal Findings:**

The established buffalo mammary epithelial cell line (BuMEC) exhibited epithelial cell characteristics by immunostaining positively with cytokeratin 18 and negatively with vimentin. The BuMEC maintained the characteristics of its functional differentiation by expression of β-casein, κ-casein, butyrophilin and lactoferrin. BuMEC had normal growth properties and maintained diploid chromosome number (2n = 50) before and after cryopreservation. A spontaneously immortalized buffalo mammary epithelial cell line was established after 20 passages and was continuously subcultured for more than 60 passages without senescence.

**Conclusions:**

We have established a buffalo mammary epithelial cell line that can be used as a model system for studying mammary gland functions.

## Introduction

There are about 158 million water buffaloes in the world, and that 97% of them (approximately 153 million animals) are in Asia. Buffaloes contribute about 15% of the total world milk supply. Buffalo milk contains higher total solids (protein, fat, minerals) of 18–23% as compared to 13–16% in cow milk. This confers advantage in the preparation of specialized cheese, curd and other dairy products [1]. With selective breeding, improved management and the establishment of more dairy herds, milk yields in buffaloes are increasing. While a plethora of information is available on mammary gland biology and lactation function in cows [2], information on buffalo mammary gland biology is scarce. The mammary gland is a complex, highly specialized tissue with diverse physiological, biochemical and immunological functions, which has evolved to provide nutrition to the neonate. The mammary gland undergoes cyclic changes of proliferation, lactation and involution with respect to the reproductive status of the animal. The structural architecture of mammary gland is made up of secretory tissue and ductular system supported by the connective tissue. The structural unit of the secretory tissue called acini is made up of secretory epithelial cells lining the lumen and myoepthelial cells surrounding the epithelial cells. The mammary epithelial cells are involved in the synthesis and secretion of milk proteins [3]. Milk protein synthesis, cell growth and differentiation are regulated by the peptide and steroid hormones [4], cell-cell interactions [5] and cell-extra cellular matrix (ECM) interaction [6]. The cellular complexity makes it difficult to dissect out the contribution of different components in the functioning of the mammary gland. In addition, the commercial value of milk has generated great interest in understanding the mechanisms of milk production and response of mammary gland to pathogenic infections.

**Table 1:** Primer sequence and reaction conditions for PCR amplification.

Gene	Accession No.	Primer Sequence(F)	Primer Sequence(R)	Annealing Temp(°C)	Length(bp)
CSN2	DQ317447	5′AATATCCAGTTGAGCCCTTTAC3′	5′CTTAGACAATAATAGGGAAGGG3′	55	294
CSN3	NM_174224	5′TGAATATCCTCACGGAGCTAAC3′	5′TGCAGAAACTGGTGTCCATAC3′	65	324
BTN1A1	NM_174508	5′ACTGATGGATCCCATATCTATAC3′	5′GTGGGATCTCCTTTGAAATGT3′	58	175
LTF	M63502	5′GATGAAGAAGCTGGGTGCTC3′	5′TGAAAGTTGCTGCCCTTCTT3′	60	240
GAPDH	NM_00124854	5′CCAAGGTCATCCATGACAACTTTG3′	5′GGTCCACCACCCTGTTGCTGTAG3′	58	498

The molecular mechanisms of developmentally and hormonally regulated milk protein synthesis had been investigated on primary culture and mammary epithelial cell lines of murine origin. Although the development and metabolism of murine and bovine mammary epithelial glands are comparable there is substantial difference in milk composition and signalling mechanism of lactogenic hormone. While β-lactoglobulin is expressed in bovine, the same is absent in rodent mammary gland. Furthermore, the main lactogenic hormone signaling pathway i.e, jak2-stat5 pathway is prominent in rodent while the same is inconsistent in bovine [7]. Moreover, the involution in ruminant mammary gland is less extensive than that of rodents [8]. The physiology of caprine mammary gland is different from bovine in mammary secretion mechanism, which is apocrine in caprine and merocrine in bovine. The mammary gland regression and decreased milk production is associated with a decrease in the number of mammary epithelial cells in caprine [9] in contrast to the loss of differentiated function and a minimal decrease in cell number in bovine [10]. Hence, an *in-vitro* model retaining the species-specific mammary gland functions is of great importance in the study of development, differentiation and involution of mammary gland. Mammary epithelial cells can be used as expression systems for production of transgenic proteins. Transgene expression of target protein in milk has several advantages over expression in prokaryotic and yeast systems [11]. Hence it is desirable to use a fully functional and transfection efficient mammary epithelial cell line as *in vitro* screening system for superior transgenes. A few immortalized mammary epithelial cell lines, induced spontaneously and by transfection of viral gene constructs have been established till recently. Spontaneously immortalized mammary epithelial cell line of bovine BMEC+H [12] and HH2A [13], ovine NISH [14], porcine SI-PMEC [15] have been established. However, only a few cell lines express lactation specific proteins. The bovine mammary epithelial cell lines ET-C [16], BME-UV [17] and MAC-T [18] have been established by stable integration of simian virus large T antigen (SV40LTA) gene to induce immortalization. However, the process of immortalization may change the physiological pathways in transformed cell lines; hence the primary cell lines are more likely to represent *in vivo* conditions, maintaining organ specific functions and signal transduction pathways [19].

**Figure 1:**
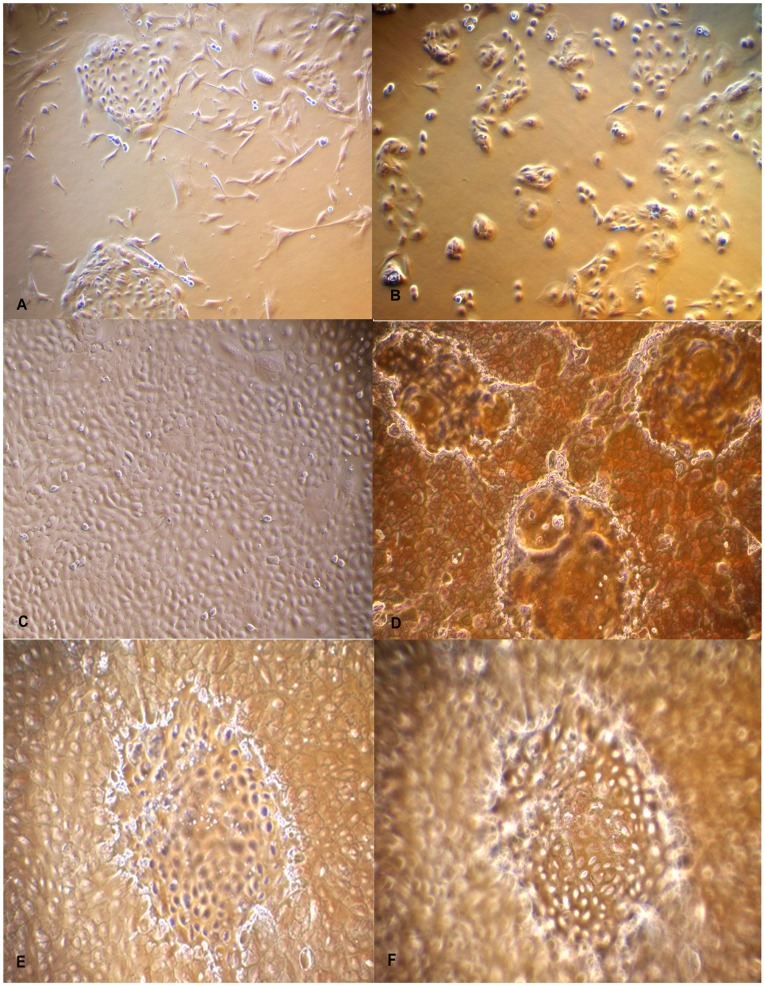
Photomicrographs of isolation and culture of Buffalo Mammary Epithelial cells (BuMECs). A: Mixed population of epithelial and fibroblast cells (×100); B: Purified BuMEC seeded at low density forming islands (×100); C: Confluent mono layer of BuMECs showing cobble stone morphology (×100); D: Post confluent stage BuMECs forming dome structure (×100); E: Phase contrast image of dome structure with focus on the monolayer (×100); F: Phase contrast image of dome structure with focus set at the top of dome (×100). The dome structure represents a raised layer of cells above the plastic substratum.

Although both cattle and buffaloes belong to Bovidae family, species-specific differences exist between these two species. Therefore, for functional studies involving buffalo mammary gland, use of buffalo mammary epithelial cell line is more appropriate than using cell lines from other related species like cattle. Till date no buffalo mammary epithelial cell line is available. Breast cancer research is an ever increasing area which needs various cell model systems for understanding the function of various biomolecules. Occurrence of mammary carcinoma is rare in large ruminants, specifically in cows and buffaloes [20]. Establishment of buffalo mammary epithelial cell line will be another model system for the research communities to study the complex phenomenon of breast cancer in general and bovine mammary carcinogenesis, in particular. In the present investigation, we report the establishment and characterization of a buffalo mammary epithelial cell (BuMEC) line.

## Materials and Methods

### Ethics Statement

No ethical approval was needed for tissue collection because the tissue was obtained from the Idgah Slaughter House, New Delhi, India and the animals were not killed for scientific research.

### Isolation and Culture of Buffalo Mammary Epithelial Cells

Buffalo mammary gland tissue was obtained from local slaughterhouse (New Delhi, India) for isolation of BuMEC. We followed essentially the same protocol used by Ahn et al. [21] for isolation of BuMEC with minor modifications. Briefly, mammary parenchyma tissue was collected from a disease-free buffalo udder after slaughter and transported aseptically to the laboratory in ice in sterile HBSS (Sigma, USA) containing 100 U/ml penicillin, 5 µg/ml streptomycin and 50 ng/ml amphotericin (HBSS-PS). The tissue pieces were trimmed of connective tissue, including fat and washed three times with HBSS-PS. The tissue was minced with sterile blade and digested with 0.05% collagenase (Sigma, USA), 0.05% Hyaluronidase (Sigma, USA) for 3 h at 37°C. The digested tissue were further treated with 0.25% trypsin EDTA (Sigma, USA), 1% Dispase (Stem cell Technologies, USA) and DNaseI (Stem cell Technologies, USA) at a concentration of 1 mg/ml for 30 min at 37°C and filtered through 40 µ cell strainer (Stem cell Technologies, USA). The filtrate was centrifuged at 80×g for 1 minute. The pellet was washed three times with phenol red free DMEM-F12 (Sigma, USA) containing 10% FBS. The cells were seeded at a density of 2×10^5^ cells/35 mm dish (Nunc, Denmark) in growth medium, which was containing DMEM/F12 supplemented with 5 µg/ml bovine insulin (Sigma, USA), 1 µg/ml hydrocortisone (Sigma, USA), 1 µg/ml apotransferrin (Sigma, USA), 10 ng/ml EGF (Sigma, USA), 10% FBS, 100 U/ml penicillin, 5 µg/ml streptomycin and 50 ng/ml amphotericin. For induction of milk protein expression, BuMECs were grown in the growth medium supplemented with 5 µg/ml Prolactin (Sigma,USA). The cells were cultured in an incubator at 37°C under 5% CO_2_. For cryopreservation, 10^6^ cells/ml were suspended in freezing medium constituting 70% DMEM/F 12, 20% FBS (Hyclone, USA) and 10% DMSO (Sigma, USA). Cell suspensions were distributed into 1 ml aliquots in cryovials and stored in liquid nitrogen. We used selective trypsinization steps to enrich the mammary epithelial cells (MECs) preferentially and remove the fibroblast cells from the primary culture. For selective trypsinization 0.25% trypsin-EDTA (Sigma, USA) was added to the confluent monolayer of heterogeneous population of cells and allowed to act for three min at 37°C. The trypsinization was stopped by adding fresh growth media, and the detached fibroblast cells were removed. The cells in monolayer which remained attached to the surface were allowed to grow by addition of fresh growth medium. The cells were subjected to 7 continuous passages for selection of homogeneous population of BuMECs. The BuMECs were routinely evaluated for sterility by growing them in antibiotic free media. The cells were also tested for incidence of mycoplasma contamination using Myco Alert Mycoplasma detection kit (Lonza, USA).

**Figure 2:**
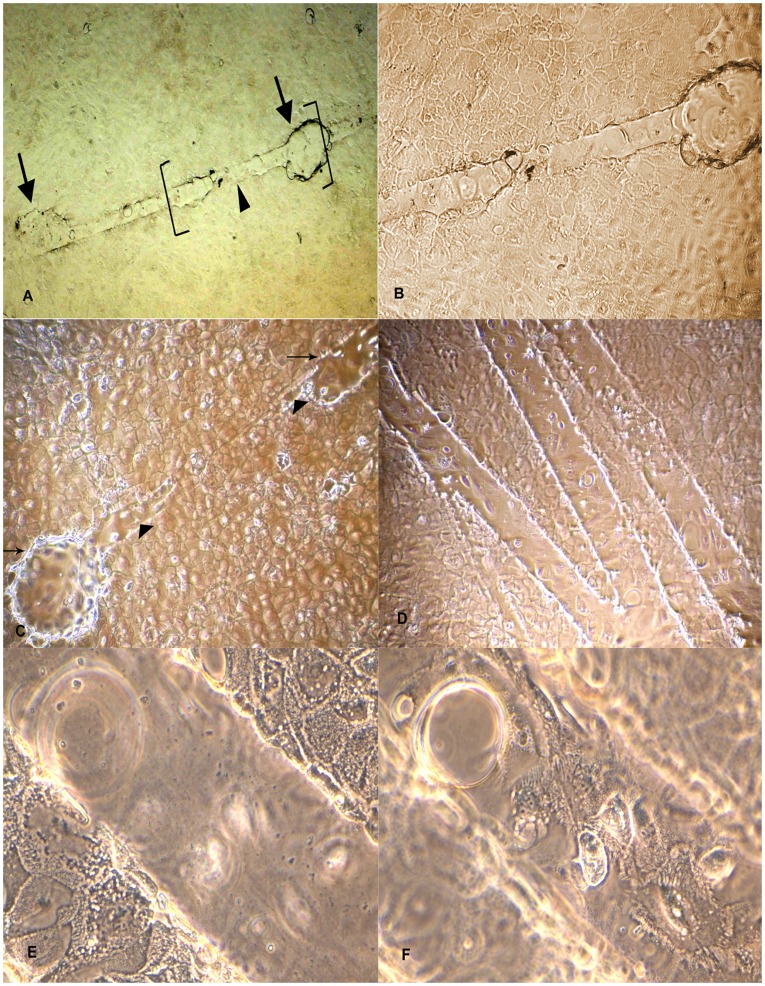
Organization of dome structure on plastic substratum. A: Development of inter-connecting structure (arrow head) between domes (arrows) in BuMECs monolayer attached to plastic substratum (×40); B: Magnified view of dome and inter-connecting structure shown in bracket in Fig. A (×100); C: Phase contrast microscopic image of an intermediate stage in the process of development of interconnecting structure (arrow head) between domes (arrow) (×100); D: Branching pattern of interconnecting structure between domes (×100); E: Magnified view of inter-connecting structure with focus on the monolayer (×400); F: Magnified view of interconnecting structure with focus on the top of the structure showing cells above the substratum (×400). This is a unique observation in BuMECs. These inter-connecting structures may represent contact mediated differentiation of BuMECs on plastic substratum.

### Growth Characteristics on Plastic Substratum

The *in vitro* growth pattern of the BuMECs was assessed by observing their doubling time at 10^th^ passage (early passage), 60^th^ passage (late passage) and frozen thawed cells (passage 25). Growth curves and doubling time were evaluated by seeding 8×10^4^ cells/well in 6 well plate (Nunc, Denmark) containing complete growth medium. Cell number and viability were determined each day in triplicate by trypan blue exclusion up to 7 days post seeding. The medium was changed every 48 h throughout the assay period. Colony forming ability of BuMECs in semisolid agar was assayed by the method described by Sun et al. [15] with minor modifications. Briefly, 1.5 ml of 0.5% base agar was prepared, poured to 35 mm dish and solidified at room temperature. About 2×10^4^ cells suspended in growth medium containing 0.5% agar were overlaid on the base agar and incubated at 37°C under 5% CO_2_. HT-29 (Human colon adenocarcinoma cell line) grown in DMEM/F12 with 10% FBS was used as positive control for colony formation. The dishes were replenished with fresh medium every 48 h. Growth and morphology of the cells were assessed every day using inverted phase contrast microscopy (Olympus 1×51, Japan) for 15 days.

**Figure 3:**
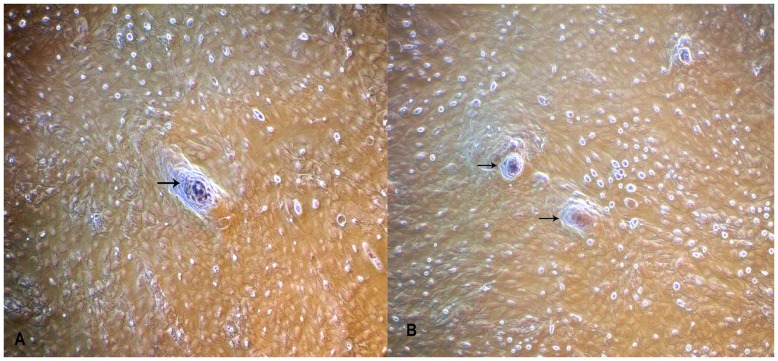
Development of papillate structures in BuMECs on the plastic substratum. A: BuMECs from passage 6 forming papillate (arrow) structure after 15 days of growth on plastic substratum (×100); B: BuMECs from passage 8 forming papillate (arrow) structures after 15 days of growth on plastic substratum (×100). Papillate structures represent a small nipple-like projection above the plastic substratum indicating differentiation characteristics of BuMECs.

**Figure 4:**
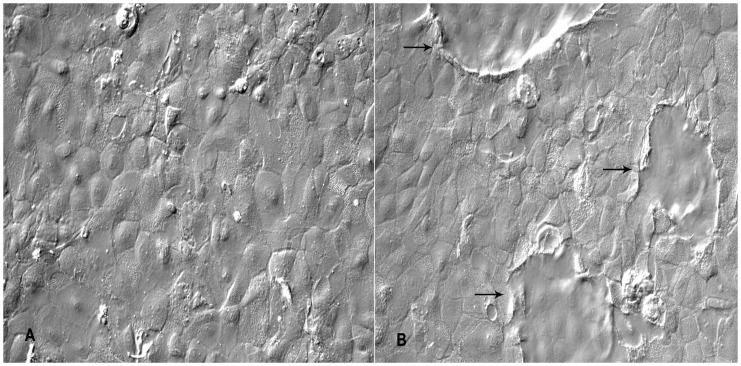
Insulin and Hydrocortisone increases dome formation in BuMECs. A: BuMECs grown in the absence of insulin and hydrocortisone (×200); B: BuMECs grown in the presence of insulin and hydrocortisone showing increased formation of domes (arrow) (×200). Results represent three independent experiments.

### Growth on Collagen Matrix

Collagen gels were prepared using bovine skin collagen Type I (Sigma, USA) following the manufacturers instruction. Briefly, 300 µl of neutralized bovine collagen type I per well at a concentration of 2 mg/ml was used to coat 24 well tissue culture plates. BuMECs at a concentration of 2×10^4^ cells in growth medium/dish was layered over the basal coating and allowed to attach for 8 h. After attachment the growth media was aspirated and overlaid with collagen solution. Cells were grown in growth medium with subsequent changes in every 48 h for up to 10 days. Morphological changes observed in BuMECs were photographed using phase contrast microscope (Nikon Ti Eclipse, Nikon, Japan). To study the cellular organization of the structures which developed in collagen matrix, the cells were stained with nuclear counter staining dye propidium iodide (500 nM) after fixing the cells with ice–cold methanol. The cellular structures were photographed using phase contrast fluorescence microscope (Nikon Ti Eclipse, Nikon, Japan).

### Senescence Associated β-galactosidase Assay (SA- β-gal)

SA- β-gal assay was performed using senescence cells histochemical staining kit (Sigma, USA) following manufacturer’s protocol. Briefly, monolayer of BuMECs at passage 15 and 60, skin fibroblast cells (senescent stage-positive control) and CHO-K1 cell (negative control) were washed with 1×PBS and fixed with 1×fixation buffer for 7 min at room temperature. The cells were then washed 3 times with 1×PBS. Following the washing, the cells were incubated for 12 h at 37°C in staining solution. Photographs of stained cells were acquired using a phase contrast microscope (Olympus 1×51, Japan). Stained cells were counted in 5 high magnification fields (×400) and the extent of senescence was expressed in percentage.

### RNA Extraction and cDNA Synthesis

Total RNA from mammary tissue, BuMECs and skin fibroblast cells (negative control) was prepared using TRIzol (Invitrogen, USA) according to manufacturer’s protocol. RNA integrity was assessed in 1.5% agarose gel electrophoresis by observing rRNA bands corresponding to 28S and 18S. Possible genomic DNA contamination in RNA preparation was removed by using DNA free kit (Ambion, USA) according to manufacturer’s protocol. Purity of RNA was checked in UV spectrometer with the ratio of the OD at λ260 and λ280 being >1.8. 1 µg of DNA free RNA was used for first strand synthesis for each sample. Primers for β-casein (*CSN2*), butyrophilin (*BT1N1A1*), κ-casein (*CSN3*), lactoferrin (*LTF*) and *GAPDH* were designed using primer 3.0 software (Table 1). cDNA was synthesized using Revert Aid First strand cDNA synthesis kit (Fermentas, USA) by reverse transcription PCR. Briefly, 1 µg RNA was reverse transcribed using RevertAid M-MuLv reverse transcriptase (200 U/µL), Rnase Inhibitor (20 U/µL), 10 mM dNTP mix (1 µL) oligo dT primers in 5×reaction buffer. The PCR reaction was performed in master cycler gradient (Bio-Rad, USA) using the following temperature–time program: 95°C for 3 min, 95°C for 30 secs, 55°C for 30 secs (CSN2)/58°C for 30 secs (BT1N1A1)/65°C for 30 secs (CSN3)/60°C for 30 secs (LTF)/58°C for 30 secs (GAPDH), 72°C for 1 min for 35 cycles and 72°C for 10 min in the last cycle.

**Figure 5:**
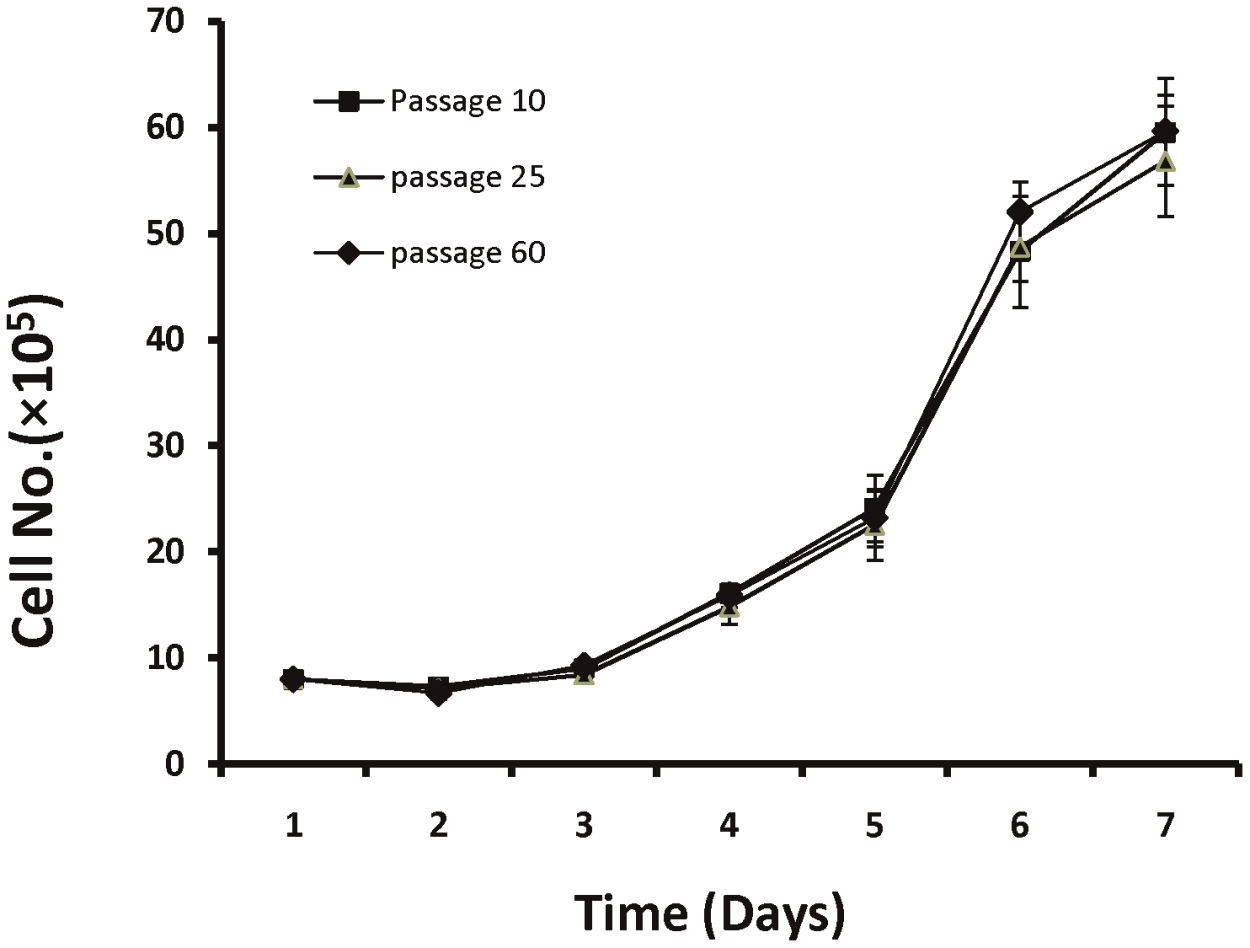
Growth curves for BuMECs at early passage (10), late passage (60) and after revival from cryopreservation (passage 25). Results are means ±SD of three independent experiments.

**Figure 6:**
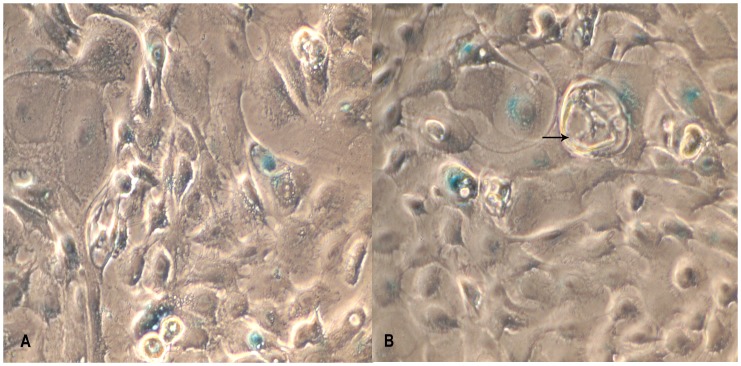
Analysis of cell Senescence–associated β-galactosidase (SA-β-gal) activity in BuMECs. A: SA- β-gal staining in early passage (10) BuMECs (×400); B: SA β-gal staining in late passage (60) BuMECs; Staining is evident from BuMEC with normal morphology and cells with vacuoles (arrow) (×400); Results represents images from three independent experiments.

### Immunocytochemistry

Cytoskeletal proteins (Cytokeratin 18, Vimentin) and Milk protein (Casein) expression in BuMECs were examined by immunocytochemical analysis. Cells were seeded at 5×10^4^ cells per well in 2 well LabTek chambered slides (Nunc, USA). Cytoskeletal protein expression was examined at day 5 post seeding by fixing the cells with ice-cold methanol for 10 minutes and permeabilized with 0.25% Triton X-100 in PBS. The cells were blocked with 10% normal goat serum for 30 minutes at room temperature. The primary antibodies for-cytokeratin 18 (Abcam, USA) and vimentin (Abcam, USA) diluted 1∶200 in PBS was added to cells and incubated for 1 h at room temperature in a rocking platform. The cells were washed with PBST 3×10 minutes. The secondary antibody, FITC-conjugated goat anti-mouse antibody (Merck, India) diluted 1∶100 in PBS was added to cells and incubated in the dark for 30 minutes on a rocking platform. The cells were washed with PBST 3×10 minutes and visualized with a phase contrast fluorescence microscope (Nikon Ti Eclipse, Nikon, Japan) using appropriate excitation and emission filters. Propidium iodide (500 nM) was used as nuclear counter stain. For a negative control experiment, the entire procedure was followed except that the primary antibody was replaced with mouse IgG as control (Santa Cruz,USA). For casein expression analysis, monolayer of BuMECs at day 10 post seeding was washed with DPBS and fixed with ice cold methanol. Non specific binding sites were blocked by 1% normal goat serum (Merck, India). Cells were incubated with rabbit anti-bovine casein antibody (Millipore, USA) diluted 1∶25 in DBPS for 1 h at 37°C. As a negative control the primary antibody was replaced with normal rabbit IgG (Santa Cruz, USA) in one of the wells. The cells were washed 3×5 min with DBPS and incubated with diluted (1∶200 in DPBS) goat anti-rabbit FITC conjugated secondary antibody (Merck, India) for 1 h at 37° followed by washing 3×5 min with DPBS. Slides were visualized with a fluorescent phase contrast microscope (Nikon Ti Eclipse, Nikon, Japan) with appropriate excitation and emission filters. DAPI (300 nM, Sigma) was used as nuclear counter stain.

**Figure 7:**
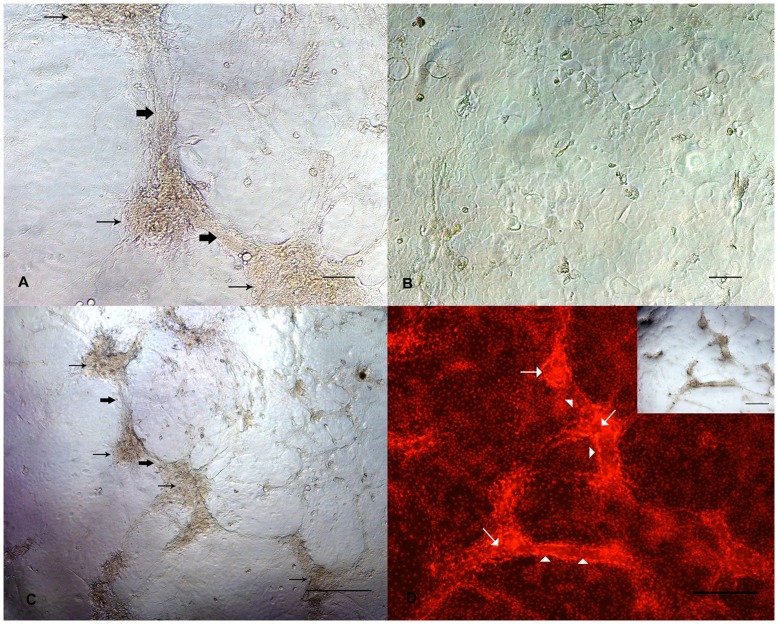
Morphological differentiation of BuMECs cultured in Type I collagen matrix. A: Phase contrast microscopic image showing development of cellular aggregates (thin arrow) and ‘duct–like’ (bold arrow) structures in BuMECs grown in attached collagen Type I matrix for four days; B: Phase contrast microscopic image of BuMECs grown on plastic substratum for four days shows no such morphological changes; C: Phase contrast microscopic image (low magnification) showing development of cellular aggregates (thin arrow) and ‘duct–like’ (bold arrow) structures in BuMECs grown in attached collagen Type I matrix for four days; D: Fluorescent image of acini-like cellular aggregate and duct- like structure in monolayer of BuMECs grown on attached collagen Type I matrix which is evident from the propidium iodide stained nucleus of the cells forming the structures (insert image show the phase contrast image of the field). Acini-like structure (arrow) constitutes a large aggregate of PI stained nuclei and duct-like structure (arrow head) showing a clear arrangement of PI stained nuclei along the sides of duct-like structure suggesting the formation of wall and lumen. Bars, A and B 100 µm, C and D 500 µm Results represent images from two independent experiments.

### Western–Blotting

Total protein was isolated from BuMECs between passages 10 and 15, fibroblasts cells (negative control) and lactating mammary tissue (positive control) using Q proteome mammalian protein isolation kit (Qiagen, USA). Total protein in the lysates was quantified by Bradford assay. Five µl of buffalo milk was mixed with SDS sample buffer and heated in boiling water for 10 minutes. An aliquot of this preparation was used as positive control for casein detection. For detection of secreted casein in conditioned medium, culture supernatants were removed and concentrated 10 fold. The lysates and culture supernatant were separated in 12% polyacrylamide gels and transferred to PVDF membrane using semi-dry transblot apparatus (GE, USA). The membrane was blocked in NAP blocker (G Biosciences, USA) in TBST for 1 h. Transferred membranes was incubated with primary antibodies of bovine casein (1∶5000), and Actin (1∶2000) (loading control) diluted in TBST and incubated overnight at 4°C. The membrane was washed 3×15 min using TBST and then incubated with diluted (1∶4000 in TBST-NAP blocker) ECL plex Cy 5 Dye conjugated secondary antibody (GE, USA) for 1 h at room temperature. The membrane was washed with TBST 3×15 min and scanned for fluorescence detection using Typhoon Trio + scanner (GE, USA).

### Chromosome Analysis

Exponentially growing BuMECs at early passage (passage 20) and late passages (passage 50) were incubated with colchicine (10 µg/ml) (Sigma, USA) for 6 hours. The cells were trypsinized with 0.25% trypsin and treated with hypotonic KCL solution (56%) for 30 minutes at 37°C. The cells were centrifuged at 200×g for 10 minutes for preparing the cell pellet. The cells after resuspension were fixed with ice-cold methanol: acetic acid (3∶1) for 30 minutes at −20°C and the same process was repeated twice. Finally, the cells were resuspended in 0.5 ml of with ice-cold methanol: acetic acid (3∶1) and dropped on to an ice-cold glass slide. The slides were dried and stained with Giemsa stain for 10 minutes, washed with distilled water and dried at room temperature. The chromosomes were visualized with phase-contrast microscope and analysed using cytovision genus software (Applied Imaging, USA) for karyotyping. A total of 50 metaphase spreads at passage 20 and passage 50 were counted, and the modal chromosome number was determined.

**Figure 8:**
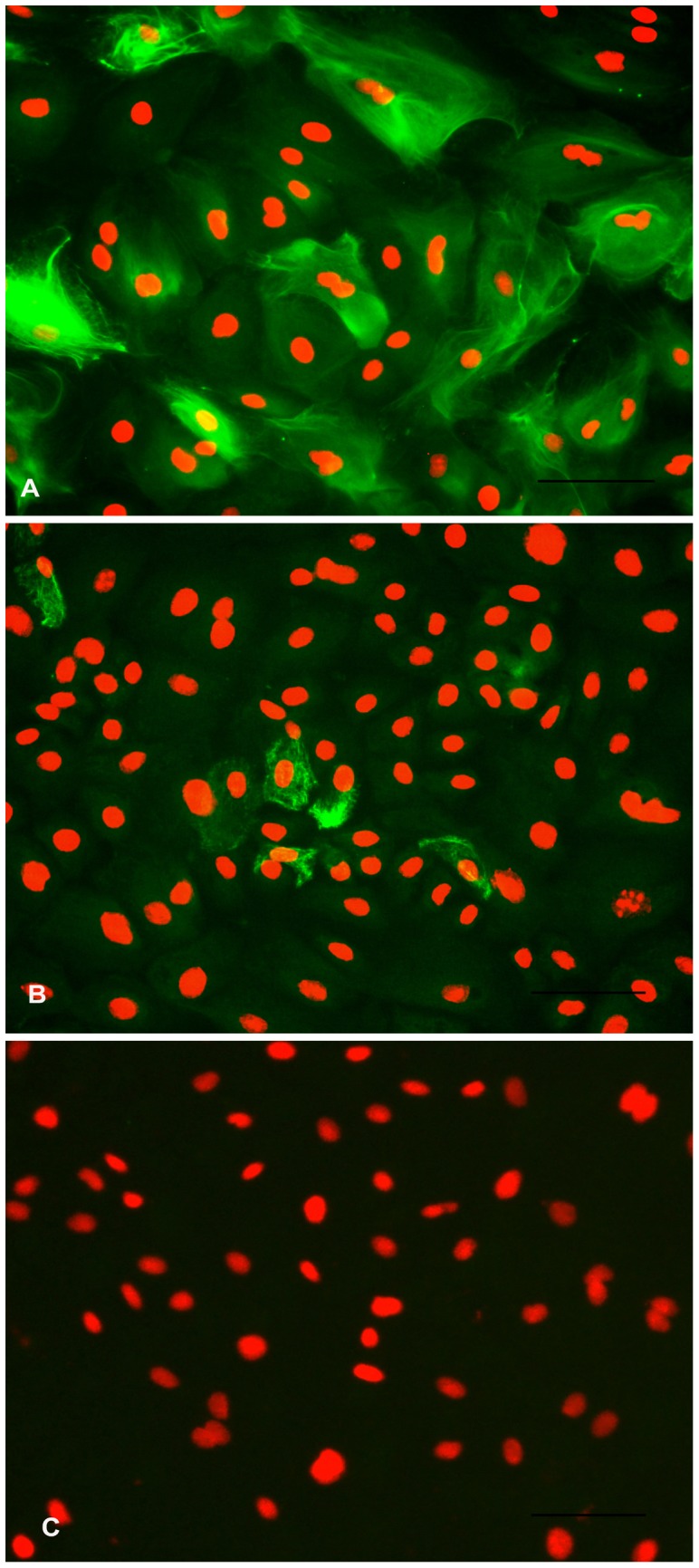
Immunostaining for cytoskeletal markers in BuMECs. A: Fluorescent image of BuMECs stained for Cytokeratin 18 showing intermediate filaments; B: Fluorescent image of BuMECs stained for Vimentin; C: Negative control with primary antibody replaced with a normal mouse IgG (Isotype control). The secondary antibodies were goat anti-mouse FITC conjugated antibody. Propidium iodide was used as a nuclear counter stain. Bars 100 µm. Results represent images from three independent experiments.

## Results

### Establishment and Growth Characteristics of BuMECs

Primary culture obtained from enzymatic digestion of buffalo mammary tissue contained heterogeneous population of epithelial and fibroblast-like cells when cultured on plastic substratum. The freshly isolated cells after 5–6 days of growth on plastic substratum developed as mixed population of cuboidal shaped epithelial cells surrounded by spindle shaped fibroblasts cells (Fig. 1–A). Further passages with selective trypsiniszation resulted in the removal of fibroblasts and yielded a homogenous population of cuboidal BuMECs. When cultured at low density BuMECs formed islands (Fig. 1–B) and exhibited typical cobblestone morphology on reaching confluency (Fig. 1–C). In early post confluency (3–5 days) dome shaped structures emerged (Fig. 1–D) which constituted a layer of cells raised above the plastic substratum. This was further confirmed by phase contrast microscopic images of domes with the objective focussed at different planes; once at monolayer (Fig. 1–E) and then on the top of dome (Fig. 1–F). Few domes in the monolayer were obsereved to be connected by unique interconnecting structures (Fig. 2–A and Fig. 2–B). Figure 2–C appears like an intermediate stage in the process of development of inter-connecting structure between the domes. The interconnecting structures formed branching patterns (Fig. 2–D). These elongated branched structures showed cellular organization similar to that of dome (Fig. 2–E and Fig. 2–F). In later stages of confluency (15 days onward) MECs formed papillate/mastoid structures (Fig. 3–A and Fig. 3–B). These structures appeared sporadically in the monolayer irrespective of the passage level of the BuMECs. Similar observations have been reported by different groups in bovine [22], [23], porcine [24] and caprine [25]. Furthermore, we observed a 4 to 5 fold increase in the formation of domes in hormone treated BuMECs in comparison to BuMECs without hormone treatment (Fig. 4).

**Figure 9:**
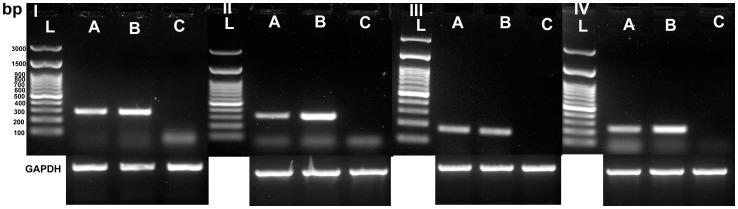
RT-PCR analysis for β-casein (I), κ-casein (II), Butyrophilin (III) and Lactoferrin (IV) in BuMECs. L: 100 bp ladder; A: BuMECs; B: Mammary Tissue (Positive control) and C: Skin fibroblasts (Negative control); Loading control represents the house keeping gene Glyceraldehyde 3–phosphate dehydrogenase (GAPDH). Results representative of minimum three independent experiments.

**Figure 10:**
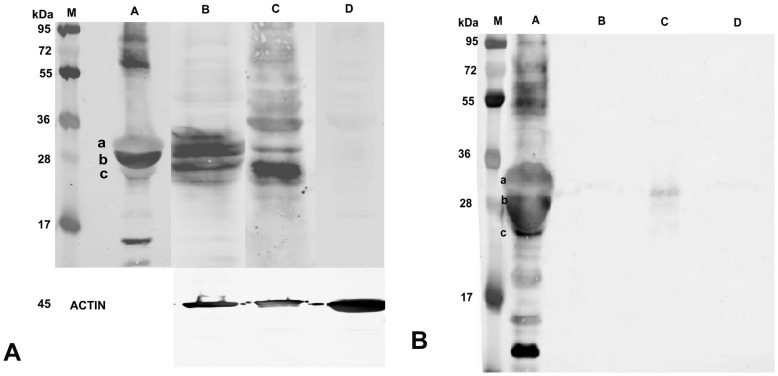
Analysis of casein expression in BuMECs. **A:**Western blot analysis for casein in BuMEC lysates, M-Protein MW standard, A-Buffalo milk, B-Mammary tissue lysate (Positive control), C-BuMEC lysate, D-Skin fibroblasts lysate (negative control) ACTIN- Loading control. **B:** Western Blot analysis for casein in BuMEC conditioned media, M–Protein MW standard, A-Buffalo milk, B-Concentrated growth medium (negative control), C-BuMEC conditioned medium, D-Conditioned media from Skin fibroblasts a; α-casein, b; β-casein, c; κ-casein in A and B.

**Figure 11:**
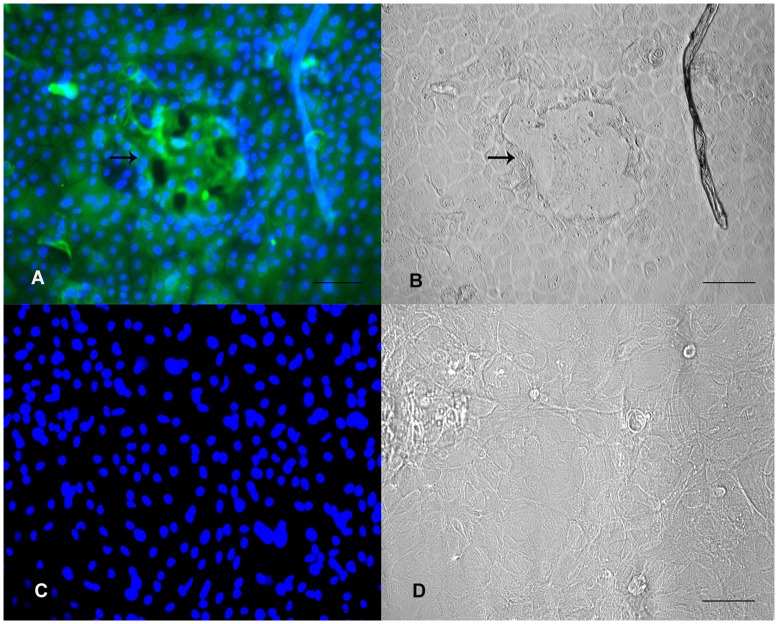
Immunostaining for casein in BuMECs A: Fluorecent image of casein producing BuMECs with relatively stronger signal from cells associated with domes (arrow); B: Light microscope image of same field as in A; C: Negative control experiments with rabbit IgG (Isotype control); D: Light microscope image of same field as in C. The secondary antibodies were rabbit anti-mouse FITC conjugated antibody. DAPI was used as nuclear counter stain. Bars 100 µm. Results represent minimum of three independent experiments.

Growth curve analysis of BuMECs on plastic substratum revealed a population doubling time of about 36–48 hours without any substantial changes between passage 10 (early), frozen thawed MECs at passage 25, and passage 60 (late passage). BuMECs reached confluency at day 6 after seeding (Fig. 5). Contact inhibition among cells was observed at post confluent stage that was evident from the floating dead cells in the medium. BuMECs showed all the characteristics of normal cellular phenotype without any sign of transformation. The non transformed state of BuMECs was further confirmed by soft agar colony formation assay. BuMECs at passage 50 did not form colonies in soft agar in the presence of hormone supplemented media after 15 days in culture. The experiment was repeated twice with BuMECs at different late passages (55 and 60) with consistent results (Data not shown). Senescence Associated β-galactosidase (SA-β-gal) assay was carried out on early passage (passage 10) and late passage (passage 60) BuMECs to assess the level of senescence. BuMECs showed staining in around 10% of cells (Fig. 6). The SA-β-gal positive cells stained blue of which the stain was mostly confined to the cytoplasm. The senescent cells showed enlarged morphology and had more number of vacuoles (Fig. 6–B). Till date we have maintained BuMECs in continuous culture for more than 60 passages without evidence of any change in growth properties and senescence.

The influence of tissue microenvironment on growth and morphology of BuMECs was studied by growing BuMEC on collagen Type I matrix. The attachment of BuMECs to collagen matrix was found to be faster than on plastic substratum. After 48 h of culture on collagen matrix BuMECs developed cellular aggregates (Fig. 7–A and 7–C) unlike on plastic substratum (Fig. 7–B). After continuous culturing for 96 h, formation of “duct-like” structures inter-connecting these cellular aggregates were observed. Counter staining of the nuclei of BuMECs with propidium iodide in these inter-connecting structures further confirmed the formation of duct-like structures with presence of lumen (Fig. 7–D). Further, our preliminary studies of growing BuMECs on surface of Matrigel (BD Bioscience, USA) revealed development of duct-like structures with apparent lateral bud (Fig. S1–A). We also observed formation of acini-like spheres when BuMECs were embedded and grown in the Matrigel for 10 days (Fig. S1–B, C and D). These data suggest that BuMECs undergo morphological differentiation in the presence of exogenous matrix.

### Cytoskeleton Expression

The BuMECs were stained with anti-cytokeratin 18 antibody to detect expression of cytokeratins, which are specific to epithelial cells and with anti-vimentin antibody to detect vimentin, which is specific for stromal cells like fibroblast cells. Almost all the BuMECs revealed strong positive staining for cytokeratin 18 (Fig. 8–A) after 5 days post seeding that confirmed their epithelial origin. Immuno staining of cytokeratin revealed intense network of cytokeratin intermediate filaments with clear intercellular tonofilament junctions. These networks of cellular structures are vital for intercellular communication and polarity [19]. In contrast a majority of BuMECs were not stained by anti-vimentin antibody except very few cells, which stained lightly (Fig. 8–B) with evidence of filament degradation. The staining in these cells was largely restricted to the periphery part of the cytoplasm. Negative control experiment with Mouse IgG isotype revealed no specific staining of cells (Fig. 8–C). The experiment was repeated three times with reproducible results.

### Milk Protein Expression

Analysis of expression of milk protein in mammary epithelial cell is important for developing optimal culture conditions and establishing the status of functional differentiation *in vitro*. The protein synthesizing property of the BuMECs was analyzed by reverse transcriptase PCR (RT-PCR), western blotting and immunocytochemistry. Expression of mRNA for *CSN2, CSN3, BTN1A1* and *LTF* were determined by RT-PCR. Robust amplification of the transcripts for *CSN2, CSN3, BTN1A1* and *LTF* was observed in BuMECs cultured in the presence of insulin, hydrocortisone and prolactin between passages 10 and 20 (Fig. 9). Western blot analysis revealed that lysate of BuMECs, mammary tissue and milk reacted positively with anti-casein antibodies while no reactivity was observed in the lysate of fibroblast cells (negative control) (Fig. 10–A). We observed casein bands of slightly low molecular weight in BuMEC (Fig. 10–A, Lane C) compared to casein bands in milk (Fig. 10–A Lane A) and mammary tissue (Fig. 10–A, Lane B). The variation in the size of the caseins in BuMECs may be due to differences in post translational modification between intracellular and secreted form of the casein which was earlier reported in mouse mammary epithelial cell line [26]. Casein secretion by the cultured BuMECs in growth medium was examined by western blotting of concentrated conditioned medium using anti-casein antibody. Weak bands (Fig. 10–B) corresponding to milk casein (Lane A) were detected in BuMEC conditioned media (Lane C). The lanes B and D represent the negative controls which are concentrated growth medium and conditioned medium from skin fibroblasts respectively. Immunocytochemistry of BuMECs showed positive staining for caseins with comparatively stronger signals in cells associated with domes (Fig. 11–A and Fig. 11–B), Negative control experiment with rabbit IgG isotypes revealed no staining (Fig. 11–C and Fig. 11–D).

### Chromosome Analysis

**Figure 12:**
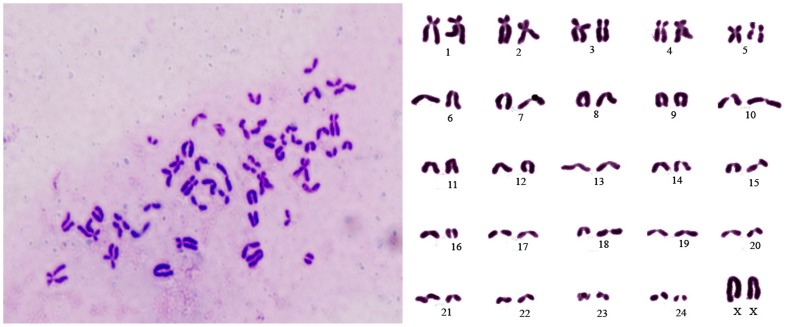
Chromosomal analysis of Buffalo Mammary Epithelial Cells (BuMEC). Representative metaphase spread and karyotype of BuMECs showing 25 pairs of chromosomes specific to buffalo (2n = 50).

Chromosome analyses of early passage (passage 20) and late passage (passage 50) BuMECs revealed normal diploid (2n = 50) chromosomes number which are specific for water buffalo [27]. Representative images of metaphase spread and karyotype are shown in Figure 12.

## Discussion

The objective of this study was to establish a buffalo mammary epithelial cell line and characterize its functional properties. With no established mammary epithelial cell line available for buffalo, this study assumes great significance because of its importance in the study of mammary specific gene functions in buffaloes and other related species. The enzymatic digestion of mammary gland tissue yielded a heterogeneous population of cells with both epithelial and fibroblast-like cells. The BuMECs showed the properties similar to bovine [21] and caprine [19] MECs which grew on plastic substratum and remained sensitive to lactogenic hormones. However, these properties were in contrast to the observations made by other groups in bovine [28], [29] and caprine [30] who reported that collagen was essential for the growth and protein production in mammary epithelial cells.

BuMECs exhibited cobblestone morphology similar to mammary epithelial cells from other species and formed islands when seeded at low density. At post confluent stages BuMECs formed dome-like structures, which appeared sporadically throughout the monolayer. It has been reported that the dome-like structure develops due to accumulation of fluid under the epithelial cell monolayer when they grow on plastic substratum [31]. This phenomenon corresponds to the cellular changes occurring *in vivo* when tubules and alveoli are developed in the mammary gland during pregnancy [32]. Formations of spontaneous dome structures in BuMECs grown on plastic substratum suggest that these cells undergo contact mediated differentiation and secrete basement membrane components. MEC lines have been reported to show dome-like structures in bovine [22], ovine (NISH) [14], human [33] and in rat in the presence of DMSO [34]. The SV40 larger T antigen induced immortalized MAC-T cells of bovine origin have also been reported to form dome-like structures when cultured on collagen [18] or when co-cultured with bovine myoepithelial cells [35]. Interestingly the dome formation in BuMECs occurred at a higher frequency when grown in medium containing insulin and hydrocortisone in comparison to the cells grown in basal growth medium. Hormone induced cell polarisation and directed secretion of proteins to basal side of MECs have been reported in mouse [36] and ovine [37]. One of a unique feature in BuMECs was the development of interconnecting structures (Fig. 2–A, 2–B and 2–C) in between domes, which further showed branching pattern (Fig. 2–D) and similar cellular organization (Fig. 2–E and 2–F) which were observed in the dome. A possible reason for this may be the extension of contact mediated differentiation of BuMECs in a defined direction from one dome to another in the monolayer. This characteristic feature of BuMECs has not been reported in other species.

Growth of BuMECs on plastic substratum revealed a population doubling time of 36–48 h (Fig. 5) representing the characteristics of normal non-transformed phenotype. Similar observations have been reported in bovine MECs [38]. However, Hu et al. [22] observed a doubling time within 72 h in MECs derived from Chinese Holstein cows. To ascertain the effect of frozen preservation on viability of BuMECs, we compared the growth characteristics of unpreserved early passage (Passage 10), frozen thawed cells at passage 25 and at passage 60. Growth curves of all the three BuMECs at different passages showed a typical “S” sigmoid curve wherein during the first three days of latent phase, the growth rate was slow. In the next three days, there was an increase in the growth rate of BuMECs followed by a steady phase. Growth pattern of BuMECs at early, late passage and frozen thawed conditions suggests that the established cell line maintain identical and favourable growth characteristics even after cryopreservation. Therefore, frozen preservation did not have any effect on the proliferation of isolated BuMECs. Similar observations have been reported in bovine [35], caprine [19] and porcine [15].

Bovine MECs have been reported to spontaneously overcome the proliferation barriers leading to immortalization [38]. To assess the proliferative characteristics and the extent of senescence in BuMECs, we performed SA- β-gal staining at passage 60. Immortalization is a process where cultured cells escape senescence and acquire the ability to grow indefinitely [39]. Around 10% of the BuMECs stained positive for SA- β-gal staining at passage 60. The staining of cytoplasm of senescent BuMECs was evident from the morphologically enlarged and flattened cells with more vacuoles (Fig. 6–B). Presence of very few senescent cells among a largely populated viable BuMECs suggests that the cells have undergone random transformation events leading to possible immortalization. Spontaneously immortalized bovine MECs have been reported to maintain normal morphology and proliferation characteristics with 10% of the cells being positive for SA- β-gal staining, which constituted non-immortal cells [40]. Classical features of senescence in Human mammary epithelial cells show flat morphology, presence of vacuoles and positive staining for senescence-associated β-galactosidase (SA-β-gal), a marker for senescence [41].

To assess the differentiating capacity of BuMECs the cells were grown on attached collagen type I matrix. The morphological differentiation of BuMECs to duct-like and acini-like structures on attached collagen gels provide evidence for their responses to microenvironment (Fig. 7). To ascertain the formation of ducts, these structures were counter stained with propidium iodide. Interestingly the acini-like structure contains more nuclei and duct-like structure shows a clear arrangement of stained nuclei along its sides suggesting the formation of wall and lumen. This observation is further supported by the development of duct-like and acini-like structures in Matrigel (Fig. S1: A–D). However, an absolute demonstration of duct-like structures would need a cross-sectional view, which otherwise remains putative. Different MEC lines from different species viz, bovine [18], caprine [19] and ovine [37], mouse [42], human [43] have been found to undergo collagen mediated morphological and functional differentiation resulting in duct and mammospheres. Integrins play a significant role in cell attachment, spreading and migration *in vitro.* Human normal MECs have been reported to form ridges and ball-like structure when collagen fibrils were added to mammary epithelial cells [44] which results due to integrin mediated morphogenesis.

Cytoskeleton expression is important in identifying epithelial cell lineage. Though the established BuMECs had epithelial morphology, we investigated the cytoskeleton protein expression specific for epithelial lineage by immunocytochemistry. Cytokeratins are intermediate filaments of epithelial cells and are important in defining the cell phenotype [45]. It has been reported that cytokeratin filaments appear as interconnected bundles in the cytoplasm. The cytokeratin network is denser around the nucleus, cytoplasmic vesicles and in the periphery of the cell where the filaments run parallel to the cell surface, which after several subcultures may reduce to the area surrounding the nucleus [46]. Cytokeratin 18 is normally associated with simple epithelium and all luminal epithelial cells of human mammary gland. Positive reaction of BuMECs with anti-cytokeratin 18 antibody indicated their luminal epithelial lineage. We further analyzed myoepithelial cells contamination at early and late passages by western blot for α-Smooth muscle Actin (SMA), which is specific for myoepithelial cells in mammary gland. We did not detect α-SMA in BuMECs, which confirmed the absence of myoepithelial cells (data not shown). Our observation in BuMECs is in line with the reported expression of cytokeratin 18 in bovine [12] and human luminal MECs [47]. In contrast, we observed very low level staining of BuMECs with vimentin. The staining for vimentin in BuMECs was atypical with filaments largely confined to the periphery of the cytoplasm and showing fragmentation (Fig. 8–B). This observation was similar to earlier report in bovine [12] and differs from caprine [19] and bovine [22] where the vimentin staining was predominantly perinuclear with filament degradation. Although the exact cause of expression of vimentin in MECs is not known it has been suggested that induction of vimentin occurs as a result of culture adaptation, such as monolayer cultivation versus three-dimensional culture and increased growth rate [48].

Casein secretion is considered as an important feature of mammary epithelial cells. Differentiation of MECs is characterized by expression of milk protein such as β-casein, whey acidic protein and milk fat [49]. Milk fat droplets are secreted from MECs by a budding process in which droplets of triglyceride formed in the cytoplasm are gradually enveloped by a layer of apical plasma membrane called milk fat globule membrane (MFGM). Butyrophilin *(BTN1A1*) an acidic glycoprotein comprises over 40% by weight of total protein of bovine MFGM [50] and is specific to mammary tissue and only expressed at high levels on the apical surfaces of secretory MECs during lactation [51]. Expression of butryophilin (*BTN1A1*) and β-casein (*CSN2*) are considered to be the response of the mammary epithelial cells to hormonal induction [22]. Amplification of the transcripts of *CSN2, CSN3, BTN1A1* and *LTF* by RT-PCR in BuMECs in the present investigation suggested that the cells were functionally differentiated having normal secretory functions. The transcripts for *CSN3* were detected consistently at all stages even in the absence of prolactin induction. Similar observation has been reported in bovine MECs where *CSN3* expression was less responsive to prolactin, and expression was observed even in the absence of prolactin. Furthermore, *CSN3* was highly expressed followed by *CSN1S1* and *CSN2* [6]. Lactoferrin expression was detected in BuMECs even in basal growth medium without hormonal supplements. This suggests that BuMEC had a normal secretory function. Similar findings have also been reported in porcine MEC model [52]. The MEC line to be used for studying mammary gland biology should have the *in vivo* properties of lactogenesis. Presence of casein in BuMECs was also confirmed by western blotting which indicated their functional differentiation. All the three forms caseins such as *CSN1*, *CSN2* and *CSN3* were observed as three distinct bands in Western blot (Fig. 10–A). We also observed casein in the conditioned medium (Fig. 10–B).

We further examined the production of casein in BuMECs by imunocytochemistry for cells grown on the plastic substratum in the presence of lactogenic hormones. Observation of positive immunostaining for casein in BuMECs (Fig. 11–A and Fig. 11–B) and relatively stronger signal for casein in cells associated with domes suggest that the BuMECs can differentiate to express lactation specific proteins such as casein. Our findings are consistent with the earlier report in caprine [19] MECs. Lee et al. [53] reported that immunostaining for β-casein in mouse MECs revealed stronger fluorescence in the cells surrounding the dome. They suggested that casein synthesis on plastic is related to topology of the monolayer and low level of casein production in cells away from the dome was attributed to heterogeneity of casein producing cells on the plastic substratum [53]. Parry et al. [54] reported a strong positive anti-β-casein antibody reaction in areas of mammary cell monolayer which became detached from glass coverslips. The presence of casein detected at mRNA and protein level in BuMECs suggests that they undergo differentiation and express lactation specific proteins in the absence of an exogenous matrix. The characteristics of BuMECs described so far suggest that they express normal cellular functions specific to mammary epithelial cells. Furthermore these BuMECs maintained normal diploid chromosome number both at early and late passage indicating that the cells maintained non-transformed lineage.

In conclusion, in the present investigation, we have reported the development of a spontaneously immortalized BuMEC line which has been maintained long-term in culture with growth and functional properties unique to MECs. This cell line can be used as a model system for functional study involving mammary cell function, genomics, transcriptomics and proteomics for understanding mammary gland biology in general and mammary biology of buffalo in particular. This can also serve as a therapeutic model for application in breast cancer research.

## Supporting Information

Figure S1 Morphological characteristics of BuMECs grown on or embedded in Matrigel. A: BuMECs grown on Matrigel develops duct-like structure with apparent lateral bud (thin arrow); B: BuMECs develop acini-like sphere when embedded and grown in Matrigel; C: Single acini-like sphere in higher magnification; D: Acini-like sphere with outgrowth (bold arrow). Bars: A, B 100 µm, C, D 50 µm.
(TIF)
