# Supplementary figures and images for: Establishment and Characterization of a Buffalo (Bubalus bubalis) Mammary Epithelial Cell Line
Source: PLoS One. 2012 Jul 9;7(7):e40469. doi: 10.1371/journal.pone.0040469 (PMC3392245; doi:10.1371/journal.pone.0040469)

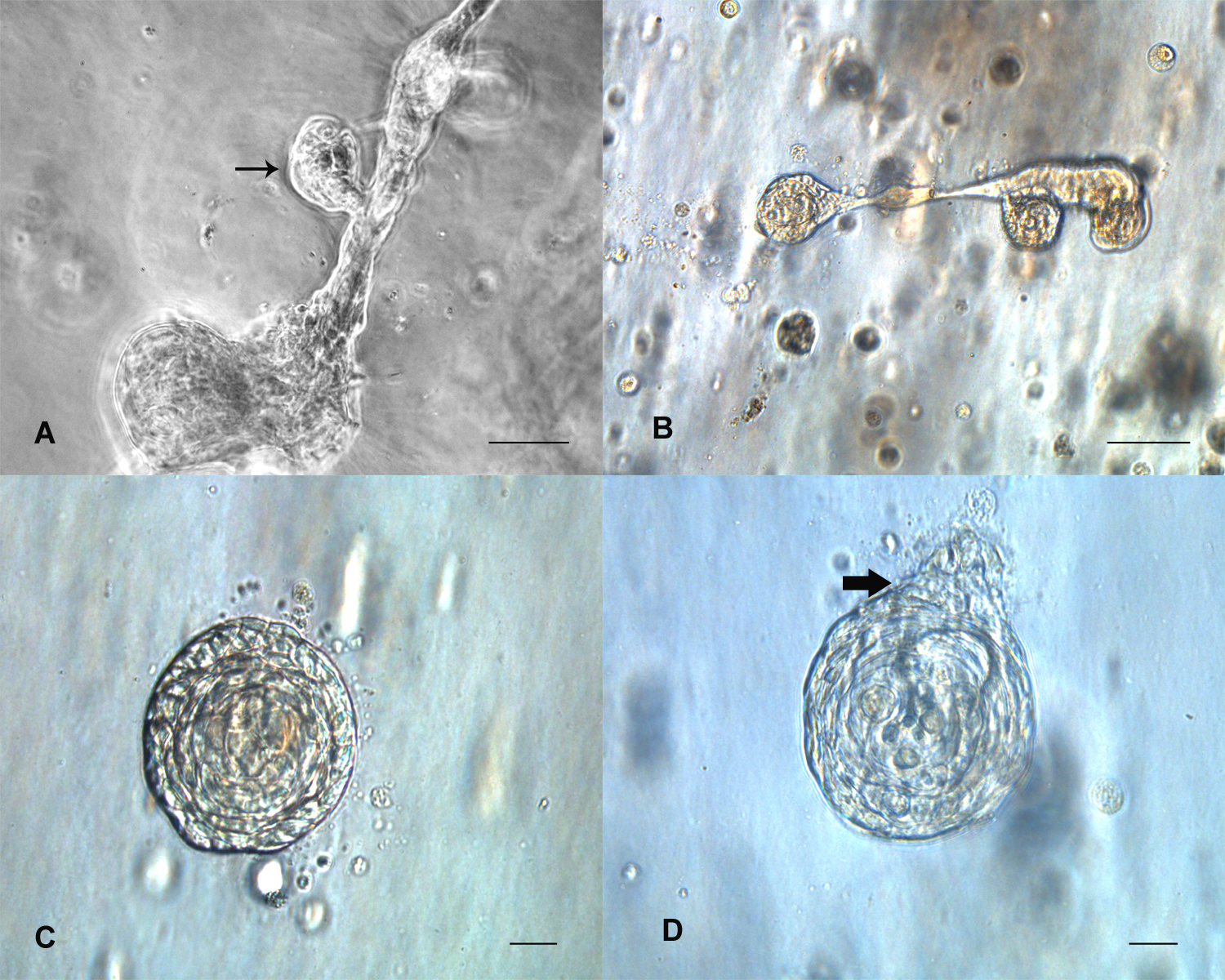

Supplement: Figure S1 — Morphological characteristics of BuMECs grown on or embedded in Matrigel. A: BuMECs grown on Matrigel develops duct-like structure with apparent lateral bud (thin arrow); B: BuMECs develop acini-like sphere when embedded and grown in Matrigel; C: Single acini-like sphere in higher magnification; D: Acini-like sphere with outgrowth (bold arrow). Bars: A, B 100 µm, C, D 50 µm. (TIF) [file pone.0040469.s001.tif]
